# Current findings in pediatric non organic feeding disorders (nofeds): the gastroenterologist point of view

**DOI:** 10.1186/1824-7288-41-S2-A61

**Published:** 2015-09-30

**Authors:** Claudio Romano

**Affiliations:** 1 Pediatric Department, University of Messina, Messina, Italy

## 

**Figure 1:**
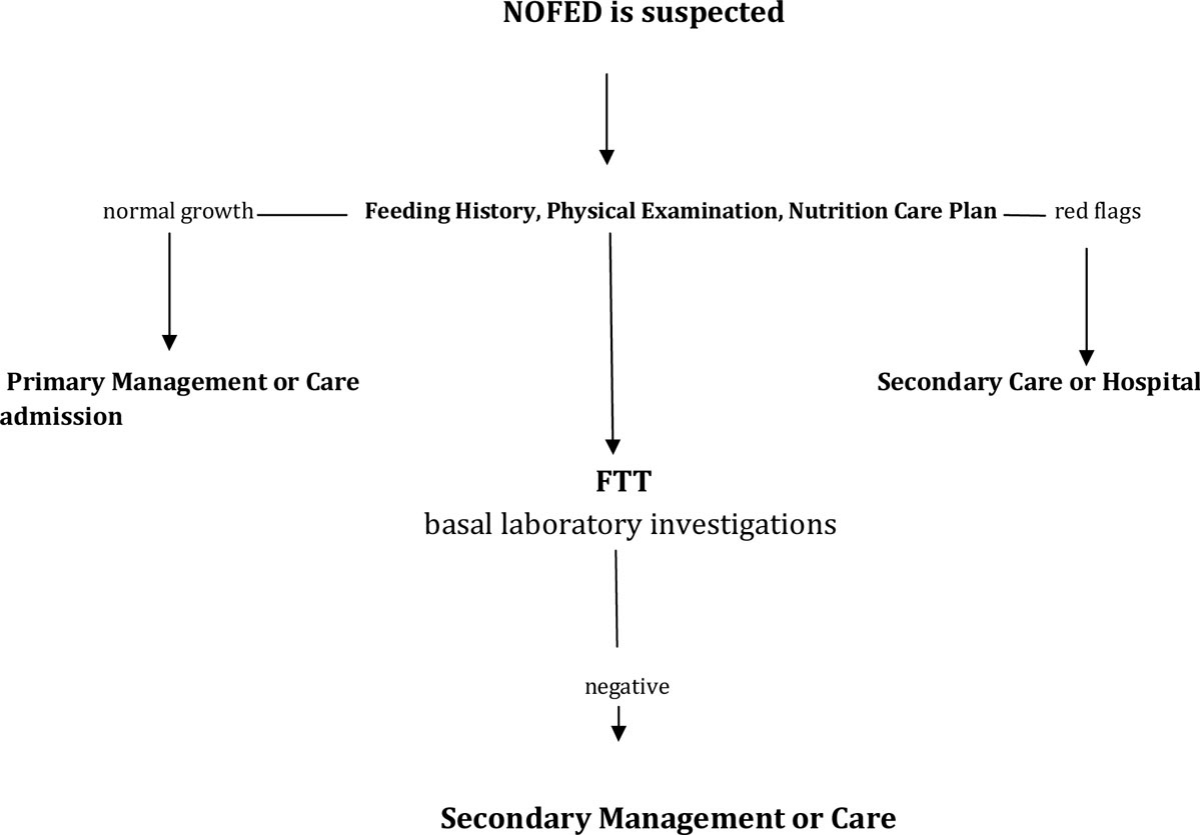
Algorithm of the NOFED

The process of taking in and swallowing food or drink involves three linked anatomical and physiological regions connected to each other both anatomically and neurologically. The non-nutritive suck is first seen in the fetus between 18 and 24 weeks. The second type of suck is the nutritive or “active feeding suck”, which is more mature and complex and is designed to deal with fluid. It is estimated that non-organic feeding disorders (NOFEDs) occur in 25% of healthy infants and 80% of young children with developmental disability [1]. Furthermore, infants born preterm and/or with a birth weight below the 10th percentile for gestational age are at high risk for developing NOFEDs. This implies that feeding disorders could be related to intrauterine growth retardation [2]. About 20-60% of parents report that their children are not eating optimally, that is, that they are picky, have food phobia, eat too little and have weight loss [3]. Several studies have suggested that only 16-30% of feeding disorders are organic and that up to 80% of cases referred to specialist pediatric services have a significant behavioral component [4]. NOFED is a formal diagnostic term used to indicate a condition in children younger than 6 years of age. It usually presents as food refusal, aversion to feeding, selective eating, low food intake or failure to thrive [5]. NOFEDs often result from multifactorial etiologies and the classification of disorders based on an organic versus non-organic dichotomy fails to provide a system that can represent the often complex interactions between medical problems, the family system and behavioral difficulties associated with feeding disorders. Some classifications are simplistic and do not help differentiate between NOFEDs and organic disorders, whereas others do not have a single set of criteria for diagnosis. The most serious complication of NOFEDs is failure to thrive (FTT) which requires a medical or nutritional approach (Figure 1) . There is often confusion, misdiagnosis and under-diagnosis with regard to NOFED and the patient is often initially diagnosed with another medical condition [6]. Differential diagnosis can include gastroesophageal reflux disease (GERD), food allergy or swallowing disorders [7]. Medical assessment should include family, social, feeding, past and current medical histories and a complete physical examination. It is necessary to emphasize the difficulties in recognizing NOFED by primary care physicians. FTT is present in 40-50% of NOFED patients correlated with a delayed diagnosis.
